# Antioxidant Therapy in Inflammatory Bowel Diseases

**DOI:** 10.3390/antiox10030412

**Published:** 2021-03-09

**Authors:** Katarzyna Dziąbowska-Grabias, Małgorzata Sztanke, Przemysław Zając, Michał Celejewski, Katarzyna Kurek, Stanisław Szkutnicki, Patryk Korga, Włodzimierz Bulikowski, Krzysztof Sztanke

**Affiliations:** 1 Department of Gastroenterology, 1st Military Research Hospital, and Polyclinic of Lublin, 20-049 Lublin, Poland; katarzyna.dziabowskagrabias@gmail.com (K.D.-G.); przemekzajac@wp.pl (P.Z.); celej88@o2.pl (M.C.); 2 Department of Medical Chemistry, Medical University of Lublin, 20-093 Lublin, Poland; 3 Department of Pneumonology, Oncology, and Allergology, Medical University of Lublin, 20-090 Lublin, Poland; katarzyna__kurek@onet.pl (K.K.); sszkutnicki@interia.eu (S.S.); 4 Department of Gastroenterology, 10ft Military Research Hospital, and Polyclinic of Bydgoszcz, 85-681 Bydgoszcz, Poland; patrykkorga@gmail.com; 5 Occupational Medicine Centre, 62-200 Gniezno, Poland; w.bulikowski@umlub.pl; 6 Laboratory of Bioorganic Synthesis and Analysis, Chair and Department of Medical Chemistry, Medical University of Lublin, 20-093 Lublin, Poland; krzysztof.sztanke@umlub.pl

**Keywords:** inflammatory bowel diseases, oxidative/nitrosative stress, antioxidant therapy, antioxidants, inflammation, reactive oxygen/nitrogen species

## Abstract

Inflammatory bowel diseases (IBD) are a group of chronic, incurable diseases of the digestive tract, the etiology of which remains unclear to this day. IBD result in significant repercussions on the quality of patients’ life. There is a continuous increase in the incidence and prevalence of IBD worldwide, and it is becoming a significant public health burden. Pharmaceuticals commonly used in IBD management, for example, mesalamine, sulfasalazine, corticosteroids, and others, expose patients to diverse, potentially detrimental side effects and frequently do not provide sufficient disease control. The chronic inflammation underlies the etiology of IBD and closely associates with oxidative/nitrosative stress and a vast generation of reactive oxygen/nitrogen species. Relative to this, several substances with antioxidant and anti-inflammatory properties are now intensively researched as possible adjunctive or independent treatment options in IBD. Representatives of several different groups, including natural and chemical compounds will be characterized in this dissertation.

## 1. Introduction

Inflammatory bowel diseases (IBD) are chronic, incurable diseases of the gastrointestinal tract, whose etiology is largely unknown. Factors that contribute to the development of IBD include genetic susceptibility, disturbed gut microflora, environmental influences, and an abnormal immune response. There are two principal types of IBD–the first is ulcerative colitis (UC), the second is Crohn’s disease (CD). The spectrum of IBD symptoms is broad and can extend beyond the digestive tract, but the most frequent symptoms include diarrhea with blood and stomach ache. It is disturbing that the incidence of UC and CD occurrence is subject to constant rise worldwide, also in the regions with previously low incidence and morbidity rates, such as Asia [1,2,3].

The primary aim of the IBD therapy is to achieve and sustain remission, as well as to influence the life quality through the alleviation of symptoms, but due to the chronic course of the above-mentioned diseases, the majority of medications applied in the therapy must be taken regularly over a long period of time. Long-term treatment significantly increases the risk of appearance of dangerous side-effect actions, which may pose a potential threat to the patient’s life. According to these facts, there is an intense search for new alternative therapeutic options without side effects or minimizing their risk [4,5,6].

Chronic inflammation is the pathophysiological process typical of IBD. In the course of inflammation, as well as other pathological conditions, such as hypoxia or infection, an increase in the intensity of oxidative stress markers is observed. Oxidative stress (OS), as well as nitrosative stress (NS), are phenomena based on the excessive formation of reactive oxygen species (ROS) and reactive nitrogen species. (RNS), which leads to the advantage of oxidants over antioxidants. One of the main places of these reactive species production is the digestive system, and the formation of them is continuous and not always harmful. These are highly reactive molecules taking part, among others, in the wound healing processes or pathogen neutralization. Nevertheless, both their excessive production and insufficient removal lead to the damage of the cell’s structural elements–proteins, lipids, or DNA. The core of the inflammatory process during IBD is ROS/RNS stimulating the excessive production of pro-inflammatory cytokines, which secondarily leads to increasing ROS/RNS production and oxidative/nitrosative stress intensification [7,8,9].

**Figure 1:**
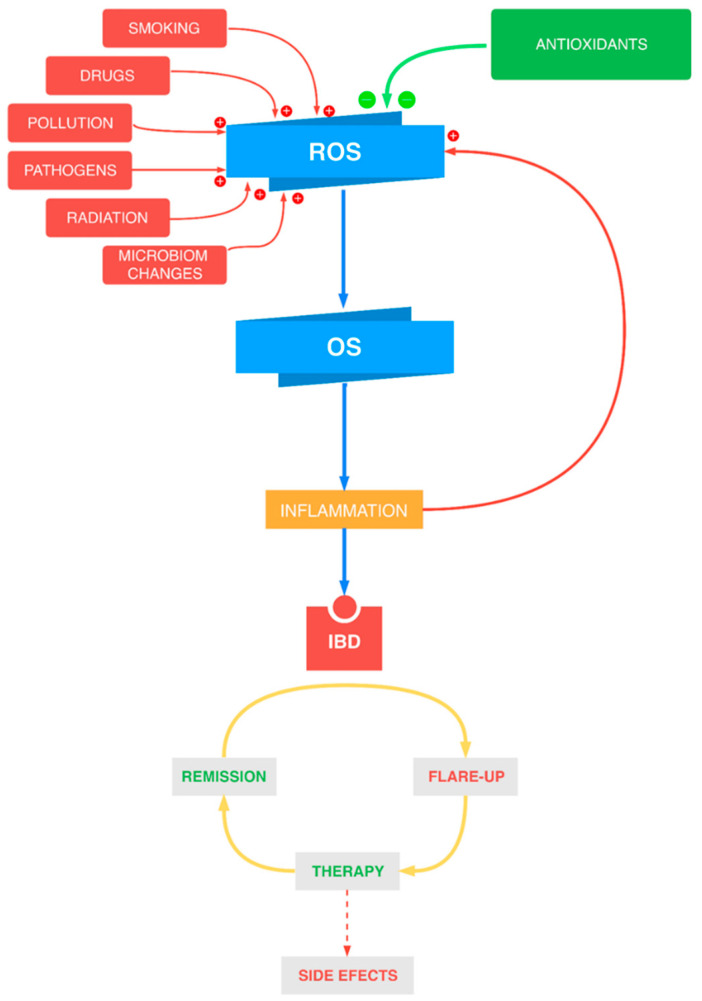
The connection between oxidative stress (OS) and inflammatory bowel diseases (IBD) and the key role of the antioxidants. ROS—reactive oxygen species.

Scientific studies confirm that oxidative/nitrosative stress plays an important role in the pathophysiology of inflammatory bowel diseases. The currently used medications applied in the IBD treatment can cause multiple side effects as well, as they do not provide sufficient control over the illness (Figure 1). Thus, new antioxidant substances are constantly being searched for. These substances are supposed to express a high safety profile as well as to influence the course of disease positively. The present dissertation aims to summarize the current scientific knowledge on the topic of antioxidants in inflammatory bowel diseases therapy.

## 2. Oxidative/Nitrosative Stress in IBD

Free radicals (FR) are particles that have at least one unpaired electron on O (reactive oxygen species) or N (reactive nitrogen species) atom. These reactive species are characterized by high reactivity and ease of chemical interaction with cellular components. They are generated during the natural course of metabolic processes, and the principal place of their formation is the mitochondrial respiratory chain. Both internal and exogenous factors can contribute to the unrestrained generation of reactive oxygen/nitrogen species and the accumulation of the oxidative/nitrosative stress. Enzymes involved directly in the reactions leading to ROS/RNS formation are, for example, myeloperoxidase, nitrogen oxide synthase, and others. Inducible nitrogen oxide synthase (iNOS), through the influence on the increased production of nitrogen oxide (NO) in the foci of the inflammatory process, plays a central role in initiating the disease process in UC and influences its intensity. The consequence of NO overproduction is increased production of tumor necrosis factor α (TNF-α), which stimulates the influx of neutrophils, activates the cascade of pro-inflammatory cytokines, and thus mimics the acceleration of ROS/RNS, exacerbation of oxidative/nitrosative stress, and intestinal damage. Another key factor contributing to the destruction of cellular structures is the activation of the transcriptional factor, e.g. nuclear factor-kappa B (NF-ĸB) [10,11].

The living organisms are equipped with the antioxidant protection system, the main purpose of which is to balance the adverse effects and protect against free radicals. It works through the enzymatic and non-enzymatic mechanisms. The non-enzymatic ones are reduced glutathione, vitamins C and E, and selenium, while the enzymatic ones are superoxide dismutase (SOD), catalase (CAT), and glutathione peroxidase (GPx). Lipid peroxidation is the first and main effect of oxidative stress because ROS act firstly on cell membranes, and the polyunsaturated fatty acids are the most susceptible molecules to radicals’ attack. This process leads to the disintegration of cell membranes, increases their permeability, infiltration, and activation of neutrophils, and—as a result—increases cell damage. The final product of lipid peroxidation is the malondialdehyde (MDA), which is one of the oxidative stress markers. Other markers of OS are the reduced activities of antioxidant enzymes, such as SOD, CAT, GPx, as well as the total antioxidant capacity (TAC) [12,13,14,15].

It has been proved that oxidative/nitrosative stress is one of the main pathophysiologic factors, initiating and contributing to the development of inflammatory bowel diseases. According to undeniable relation of the aforementioned phenomena, the intense search is being conducted to find the substances which have both antioxidant and anti-inflammatory properties and which could be used in the therapy with the hope of minimizing the side effects, improving the quality of patients’ life and, perhaps, allowing also the reduction or total replacement of essential drugs. This article presents synthetic (Table 1), natural (Table 2), and micronutrient (Table 3) antioxidants that due to their properties and promising research results, may be useful in the therapy of IBD, as supportive or independent treatment options (Table 1).

## 3. Antioxidant Therapy in IBD

### 3.1. Synthetic Antioxidants

Synthetic antioxidants used in IBD therapy include medications, hormones, enzymes, and other biochemical substances that are presented in Table 1.

#### 3.1.1. Inhibitors of 3-Hydroxy-3-Methylglutaryl Coenzyme A Reductase

Statins are inhibitors of 3-hydroxy-3-methylglutaryl coenzyme A (HMG-CoA) reductase. This group of drugs has an antioxidant effect and is widely used in pharmacological treatment due to their hypolipidemic properties. In clinical practice, especially in cardiovascular diseases, their influence on other processes is used, for example in the prevention of atherosclerotic tissue formation and its stabilization in the blood vessels. Retrospective research reveals that taking statins prevents both UC and CD, especially in older age groups. However, in patients diagnosed with IBD, the risk of colorectal cancer is significantly lower, as is the probability of hospitalization or surgical intervention. It has also been observed that patients with IBD taking statins less frequently require biological treatment or immunosuppressive drugs [16,17,36].

The statins most frequently analyzed in animal models are rosuvastatin, simvastatin, and atorvastatin. In addition to the known antioxidant and anti-inflammatory activities of statins, new properties are systematically being discovered, e.g., inhibition of apoptosis and modulation of the immune system. The research reveals that rosuvastatin efficiently prevents the effects of colon inflammation caused by dextran sodium sulfate (DSS). It is expressed by a reduction in damage at the histopathological level, preventing shortening the bowel length and body weight loss, as well as the significantly lower DAI (disease activity index), assessing the clinical activity of the disease. The level of pro-inflammatory cytokines and granulocyte colony-stimulating factor in the blood is also decreased. Rosuvastatin lowers the expression of oxidative stress markers in the tissue and also inhibits the apoptosis process, e.g., by the reduction in the protein level of enzymes from caspase group. Simvastatin in the experimental rat model of colitis has a similar alleviating effect in terms of clinical symptoms and histopathological lesions. Both simvastatin and rosuvastatin effectively increase the antioxidant capacity by raising SOD activity and reduced glutathione concentration and lowering the MDA level. They also reduce levels of inflammatory markers. Comparing the effect and the potency of simvastatin and rosuvastatin, the research reveals the advantage of rosuvastatin in an animal model of colitis [18,19]. The ability of statins to suppress the immune system response has been the subject of intense research in recent years, and therefore, in the future, they may be used to treat autoimmune diseases, including IBD. The mechanism of action of HMG-CoA inhibitors seems to be very promising and can be used in the therapy of many diseases, including also IBD. Nevertheless, it requires further research [20]. 

#### 3.1.2. Angiotensin-Converting-Enzyme Inhibitors

Angiotensin-converting enzyme inhibitors (ACEI) constitute a group of drugs lowering the blood pressure, also expressing antioxidant and anti-inflammatory properties. The influence on colon inflammation is the subject of intense research on many medicines in this group, including telmisartan, captopril, and valsartan.

In animal models of colitis, studies have been carried out on the effect of the way of administration of telmisartan (TLM) on individual parameters of the antioxidant system in order to determine the safest route of administration–oral or rectal. The research reveals that the anti-inflammatory properties of orally administered TLM are manifested by faster completion of the early, acute phase of the pathological process in the intestine. It has been proved by discovering the lowered levels of TNF-α, MPO, MDA, NO, and increased level of anti-inflammatory cytokine IL-10 in biopsy material. The study reveals that TLM reduces the mRNA level and proteins of the key factors promoting the inflammatory process, such as NF-κB, cyclooxygenase-2 (COX-2), iNOS. Telmisartan also reduces damages on the macroscopic and histopathologic level, as well as alleviates the symptoms of the diseases measured on the DAI scale. In addition to anti-inflammatory properties, TLM also enables adequate protection against free radicals by strengthening intestinal defense mechanisms–it increases the levels of GSH and TAC and the activity of antioxidant enzymes such as SOD and GPx. On the other hand, rectal administration of TLM as a retention enema in increasing the dose maximizes the local effect on the mucosa. Additionally, it significantly reduces the risk of serious side effects, which can be used especially in the left-sided form of UC in people. Moreover, TLM modulates the process of apoptosis. The relatively few possible side effects, long-term presence in the blood and favorable price are some of the features of TLM that distinguish it from other representatives of this group. As a result, TLM remains of interest to researchers as the main or additional drug in the treatment of inflammatory bowel disease [21,22,23]. Further examination compares the effect of captopril and valsartan administered prophylactically before the induction of chemical intestine inflammation or as a therapy in the control group. There are no important differences between these two pharmaceutical substances. Nevertheless, both exhibit powerful anti-inflammatory properties and enhance the antioxidant defense. Both captopril and valsartan reduce the micro- and macroscopic damage to the intestine. It has also been proved that they exhibit the immunomodulatory effects, and their combined implementation with drugs used in IBD therapy, which have the same mechanism, may lead to a reduction in the doses necessary to achieve a therapeutic effect [24]. 

#### 3.1.3. Melatonin

Melatonin (MEL) is an organic substance, synthesized mainly in pinealocytes, but other organs, such as the retina and the ovary, also have the ability to produce this natural hormone. The reduction in the intensity of oxidative stress by MEL has been proved in scientific studies. Due to its high antioxidant capacity, it remains the focus of scientists. It has unique and intriguing properties that prove its uniqueness in comparison to other antioxidants. First of all, it is soluble both in water and lipids. Thus, MEL easily penetrates cell membranes and reaches all cellular compartments, but simultaneously is selective towards mitochondria. It is a relatively safe substance, the described side effects of which are usually harmless. MEL is also characterized by the intensity of action, its antioxidant properties, compared to other known antioxidants, are more intense than those of vitamins C and E [37,38]. MEL directly diminishes the level of free radicals, which together with its metabolites, start the cascade capable of neutralizing up to 10 oxygen free radicals. Moreover, it can bind heavy metals (e.g., iron), and through this, it prevents the formation of ROS. MEL strengthens the antioxidant defense mechanisms of cells by increasing the activity of enzymes such as superoxide dismutase, catalase, and others. It has also immunomodulatory properties and limits the production of pro-inflammatory cytokines and enhances the formation of anti-inflammatory cytokines (such as IL-10) [39,40,41].

Many valuable conclusions have been drawn based on the examination of patients suffering from UC, being in remission, who had been administered orally, apart from mesalazine, 5 mg of melatonin a day for 12 months. The clinical activity of the disease was marked in Microscopic Colitis Disease Activity Index (MCDAI) score. In addition, the severity of the inflammatory process was monitored with CRP and hemoglobin levels. Furthermore, the focus has been shifted to the fact that the levels of anxiety and depression as mental disorders often accompany IBD. According to the group taking a placebo, the improvement has been observed regarding all marked parameters. Based on the data analysis, it was concluded that MEL can be used as an additional therapy in maintaining remission in ulcerative colitis [25]. 

Antioxidant and anti-inflammatory effect of MEL is observed in research on experimental animal models. The result of melatonin activity via different ways of administering has been compared, using intraperitoneal injection (IP) and a rectal route in the form of specially designed gel formula including MEL, which, thanks to its qualities, was intended to adhere tightly to the places affected by the disease process. Tissue material from rat intestines has been examined to assess the oxidative stress markers (MDA, NOx, and GSH levels). It has been proved that melatonin administered systemically acts more effectively on lowering MDA level. Both IP administering and the gel form result in the lowering of the NOx level, but none of them affects the GSH level. No significant advantage of gel formula over IP administering has been proved [26]. Other examinations on rodents reveal that melatonin prevents weight loss and prolongs the lifetime in the process of colitis, as well as lowering the degree of histopathologic damages. It also reduces the level of pro-inflammatory cytokines in blood, such as interleukin-1β, -6, -17, TNF-α, and interferon gamma, and the activity of MPO in tissue material. Currently, research is being conducted concerning the melatonin activity mechanisms, and new findings reveal that other ways than stimulating melatonin receptors are possible because its anti-inflammatory effect is also present while MT receptors are blocked by antagonists (e.g., luzindole) [27,28]. MEL significantly raises the total antioxidant capacity measured in plasma. Additionally, the analysis of the variety and composition of the intestinal microbiome in the group of mice with artificially induced IBD receiving melatonin shows, that it raises the abundance of bacteria *Firmicutes*. New scientific research shows that dysbiosis based on the lowered variety of bacteria and reduced amount of bacteria belonging to *Firmicutes* and increased amount of *Gammaproteobacteria* in the digestive tract is related to the etiology of IBD. According to this fact, the ability of melatonin to modify the intestinal microflora seems to be promising and shows another advantage of its application, apart from antioxidant and anti-inflammatory activity in IBD therapy [42,43].

#### 3.1.4. N-Acetylcysteine

N-acetylcysteine (NAC) is a derivative of L-cysteine, which has a significant influence on the oxidative stress phenomenon in the form of hampering the production of free radicals, increasing the activity of antioxidant enzymes, and decreasing the expression of heat shock proteins, which are OS markers. NAC is converted into cysteine, and in intestines acts as a substrate for the production of reduced glutathione. Its anti-inflammatory activity is well-known, and it is based on lowering the level of cytokines necessary for the development of an inflammatory process, e.g., TNF-α. NAC prevents the activation of NF-κB, which is a transcriptional factor driving the cycle of chemical reactions resulting in the activation of pro-inflammatory cytokine genes through positive feedback. NAC also contributes to the increase in the level of a chemical energy carrier, which is ATP, inhibits the apoptosis process by affecting caspases, and also promotes the processes of proliferation, development, and regeneration of intestinal cells. Furthermore, it contributes to the tightening of the intestinal barrier by influencing the proteins of claudin and occludin groups. NAC has a positive effect on the intestinal microflora, and is of significant importance according to connecting dysbiosis with IBD etiology [44,45]. 

Interesting conclusions have been drawn in the study analyzing the effect of the combined action of NAC and mesalamine (which is one of the primary drugs in IBD therapy) administered rectally in chemically-induced colon inflammation in rats. It turned out that the treatment with combined application of both substances had a better effect on parameters such as iNOS, COX-2, prostaglandin E2 than using each of the drugs separately. This suggests the possibility of using NAC as a drug supporting the treatment of inflammatory bowel disease, in addition to those used so far [29].

#### 3.1.5. Modified Superoxide Dismutase

The human organism has been equipped with a broad spectre of mechanisms preventing oxidative stress. Among the enzymes with strong antioxidant properties is superoxide dismutase (SOD). Nevertheless, it has no application in diseases connected with a high level of OS, because it is an enzyme with a short half-life and is characterized by a lack of stability in the gastrointestinal tract. Genetic engineering comes to the rescue, thanks to which it was possible to achieve recombinant bacterial strains acting as SOD donors—for example, a recombinant strain *Lactobacillus fermentum*, which on a mouse IBD model causes the improvement within the scope of clinical symptoms and reduces the mortality. Its influence on oxidative stress is expressed by the inhibition of lipid peroxidation and the reduction in MPO level in the intestine. The inflammation in a gut is alleviated by reducing the cytokine synthesis, e.g., IL-1β, IL-8, and the activity of transcriptional factor NF-ĸB [30].

Clinical trials devoted to the influence of lecithinized superoxide dismutase (PC-SOD) are being conducted in patients suffering from respiratory diseases such as interstitial pneumonia and in patients with ulcerative colitis. This new form of SOD removes the restrictions of superoxide dismutase when it comes to the duration length of its activity and the half-life. PC-SOD administered intravenously in the dosage of 40 or 80 mg a day for four weeks causes the clinical improvement within the scope of a number of stools, the presence of blood in feces, lesions of the mucous membrane in the intestine, and the general medical evaluation (UC-DAI score). The study revealed that PC-SOD might cause specific adverse effects, but they are harmless (i.e., nausea, the feeling of being unwell), and its presence does not depend on the administered dosage of the drug. According to the lack of additional benefits while administering a higher dosage and with comparable effectiveness, an optimal dosage has been determined in the amount of 40 mg/day. Scientific research reveals that the lack of additional effect in higher dosages of PC-SOD comes from the accumulation of hydrogen peroxide, and concurrent supply of catalase, which degrades hydrogen peroxide to oxygen and water, restores the activity effect of high dosages of PC-SOD. Administered intravenously in mice with intestinal inflammation induced with DSS causes the reduction of histopathological damages and affects the lowering of ROS level [31,32,46]. 

#### 3.1.6. Propionyl-L-Carnitine

Propionyl-L-carnitine (PLC) is a derivative substance of L-carnitine, which presence is required for the transport of fatty acids in the mitochondrial matrix. It is attributed to the ability to prevent the destructive influence of oxidative stress. It reduces damages arising due to the hypoxia of tissues and related to the blood flow in the process of reperfusion in organs such as heart, kidneys, or liver. By restricting the production of ROS and anti-inflammatory activity, PLC influences the healing processes positively and counteracts the damages on the vascular endothelium, which is also observed in IBD [33,47,48]. The study of Merra and colleagues [34] has been conducted in patients suffering from UC and CD with the mild or moderate course, in which 2 g/day of PLC were administered orally for four weeks. It has been proved that PLC causes the improvement within the scope of clinical symptoms, endoscopic and histopathologic image, especially in ulcerative colitis, whereas the influence on Crohn’s disease requires further examination [34]. Clinical study of the second phase conducted concurrently in many centers compared the effect of 1 g and 2 g dosages of PLC administered orally with placebo in group suffering from a mild or moderate form of ulcerative colitis. It has been proved that the application of higher dosage does not lead to achieving an additional therapeutic effect. The PLC showed an excellent safety profile and the observed adverse effects were mild and mainly related to the digestive system. In one case the patient has been noticed an increased heart rate. The best outcome has been achieved in patients with mild UC, the improvement was observed both clinically and endoscopically in comparison to the patients receiving placebo. Based on this examination the conclusions have been made that PLC may be used as an additional therapy, but further testing is compulsory in order to determine appropriate dosage and to learn extensively about the potential adverse effects [35].

### 3.2. Natural Antioxidants

Polyphenols constitute a vast group of various organic substances present in a natural way in plants. They are described to have antioxidant, anti-inflammatory, and immunomodulatory properties. The analysis of scientific data reveals that diets rich in polyphenols may alleviate the course of diseases, which pathogenesis is related to the excessive production of ROS [49,50]. Polyphenols and other substances of plant origin with antioxidant properties are presented in Table 2.

#### 3.2.1. Resveratrol

The activity of resveratrol (RSV) as an antioxidant commonly present in fruit (especially in grapes) has been tested mainly on experimentally-induced IBD models in rodents. Prophylactic intraperitoneal administration of RSV for five days before inducing colitis in rats reduces the damages on the histological level, reduces the intensity of the lipid peroxidation process marked via lowering MDA level, and strengthens the antioxidant protection system by raising the activity of GPx enzyme. The study conducted on a rat model of Crohn’s disease reveals that RSV has anti-inflammatory properties and it restricts fibrosis. It lowers the mRNA level of pro-inflammatory cytokines in the intestinal tissue, such as IL-1β, IL-6, and TGF-β1 and it reduces the mRNA level of procollagen I and III and IGF-1. Anti-inflammatory activity of RSV is also revealed by hampering the production of cell adhesion molecules [51,52,53]. Similar results within the scope of antioxidant and anti-inflammatory activities of RSV are proved on studies in humans. For six weeks the patients with diagnosed ulcerative colitis were administered 500 mg of RSV a day orally. It occurred that RSV in comparison to placebo causes an increase in SOD activity and total antioxidant status values and a decrease in MDA level in blood. It also influences positively the clinical activity of disease, reducing its scoring system in the DAI score. Moreover, RSV improves the life quality of patients. Decreasing the activity of the inflammatory process has also been noticed, expressed by CRP and TNF-α reduction in plasma. The results are highly promising. Nevertheless, further research is vital concerning the determination of optimal dosage or the way of administration for RSV [54,55].

#### 3.2.2. Curcumin

According to copious properties, among other things antioxidant, anti-inflammatory, and hampering the carcinogenesis, curcumin is being examined in the field of its application in prevention and treatment of many diseases with inflammatory etiology over the recent years. It has been used for ages in folk medicine. It is obtained from rhizomes of *Curcuma longa*, and it is very prevalent especially in Asia [73,74].

Many scientific studies have shown the curcumin’s activity in animal models of colitis and seem to be promising when it comes both to the prevention and the treatment of inflammatory diseases of intestines. Curcumin impairs the intensity of the disease process marked in DAI score, reduces the damages on the histopathological and macroscopic levels, hampers the inflow of neutrophils to the diseased parts of the intestine measured by the MPO activity as well as lowers the intensity of the lipid peroxidation process. The mechanisms of the action are still examined intensely. One of them may be the influence on hampering the activation of transcriptional factor NF-κB and STAT3 and, related to it, the expression of proteins (among other things COX-2 and iNOS). Their excessive stimulation has a direct connection with the development of inflammation and promoting the process of carcinogenesis, whereas using the drugs which inhibit these factors acts chemopreventive. The influence of curcumin on hampering the process of apoptosis has also been described [56,57,58,59,75].

The studies focused on curcumin’s influence on the course of UC in people unanimously show that it causes the reduction of clinical symptoms of the disease, increases the percentage of patients achieving remission, as well as improves the image measured in the endoscopic examination. The most frequently analyzed is the activity of curcumin as an addition to mesalamine, standardly used in therapy, comparing its effect to mesalamine with the addition of placebo. Curcumin may be administered orally in the dosage of 3 g a day or in the form of retention enema. Concurrently, curcumin is characterized by a relatively good safety profile, and the advantage of a topical therapy over the oral one may be based on the possibility of triggering the dyspepsia by oral administration [60,61]. Analyzing the clinical examinations conducted hitherto in the amount excessing 40, none of them revealed the severe adverse effect of curcumin. Taking this fact into consideration, as well as the low price of this medicament and encouraging results of studies, curcumin should be taken into account as a possible additional therapy of IBD in the future. Nevertheless, there is a need for further clinical trials to determine the most appropriate dosage and the way of administration, in order to formulate the specific recommendations [76,77,78]. 

#### 3.2.3. Quercetin

Quercetin (QCT) is a chemical substance of plant origin belonging to the group of flavonols. According to the ability to minimize the destructive results of oxidative stress, described in numerous scientific publications, the influence of QCT on diseases related to OS, including IBD, is being studied.

Numerous investigations on animal models of ulcerative colitis provide data concerning the application of this natural substance in the therapy of IBD. Quercetin administered orally in increasing dosage from 25 to 100 mg/kg for 11 days influences the course of disease significantly, apart from the lowest dosage. It is manifested by lower body weight loss, reduction of intensity of bleeding from the rectum and alleviating the damages in the intestine on the macroscopic and biochemical levels. QCT lowers the activity level of MPO in the mucous membrane of the large intestine, it boosts the level of GSH, diminishes the intensity of lipid peroxidation and damages related to the oxidative stress by influencing the level of nitrites and nitrates. Other studies on the animal models show that one of the QCT activity mechanisms is lowering the expression of TNF-α. The positive influence of intestinal macrophages’ ability to destroy the bacteria is also described. Moreover, it contributes to restoring the appropriate balance between the organism and its intestinal microflora [62,63,79]. New research concerning the influence of QCT on the transportation of 5-amino-salacylic acid (5-ASA) by cell membranes shows that using QCT as an additional therapy may contribute to the reduction of dosages of sulfasalazine vital for therapeutic effect, achieving at the same time a better control over the disease course with concurrent minimization of adverse effects [80].

#### 3.2.4. Catechins

Catechins are natural organic substances belonging to the group of polyphenols, present in abundant amounts in tea, especially in green tea. The representatives of this group, according to their potential therapeutic properties in IBD, are given full attention, and the most frequently described are especially epicatechin (EC) and epigallocatechin-3-gallate (EGCG). Catechins are characterized by the antioxidant activity, and the ability to alleviate the inflammatory process’ intensity as well as to hamper the process of neoplastic cells’ growth [81,82].

The research conducted in patients affected by a mild or moderate form of UC, whose response to the treatment applying 5-ASA or azathioprine was not sufficient, provides encouraging information. In patients receiving EGCG orally in the dosage of 400 or 800 mg a day, in comparison to the group receiving placebo, after 56 days the lower activity of the disease marked in DAI score has been observed as well as the higher percentage of remission [64]. 

In vitro tests reveal that EC influences the lowering of the intestinal mucous membrane’s permeability caused by TNF-α, which process is crucial in developing the disease. It prevents the dysfunction of cellular connections and, thanks to this, provides the tightness of a cellular barrier. The influence of EGCG on reducing the level of IL-8 and NO lowering the expression of iNOS and COX-2 have also been described [65,83]. 

The research on mice models of ulcerative colitis reveals that EGCG improves the clinical course of UC, causes weight loss to a lesser extent, and lowers the risk of death as a result of the disease. It also reduces the damages in the large intestine marked histopathologically, such as ulceration or inflammatory infiltration. It reduces the MPO activity and the MDA level in the intestinal tissue as well as boosts the expression of antioxidant enzymes such as SOD and GPx. Moreover, EGCG lowers the expression of mRNA of pro-inflammatory cytokines such as IL-6 or TNF-α, and it reaches its effects, among other things, by influencing the NF-κB pathway. Scientific reports within the scope of affecting the body mass are ambiguous. Some of them provide that it is necessary to remain cautious in applying catechins in IBD treatment, because apart from the strong anti-inflammatory properties they may cause the hampering of digestion and absorption of some nutrients, through which it can lead to the more intense body mass weight loss. This may have a positive effect in obese patients, but may be detrimental in malnutrition, which is often present in IBD. In IBD treatment, it is vital to apply the iron preparations according to the anemia imitatively ascertained to IBD. The research shows that EGCG in the environment abounding in iron may act as a pro-oxidant and contribute to boosting the ROS level, which may cause exacerbation of disease. According to the ambiguous research results, it is vital to conduct an in-depth analysis of catechins’ activity depending on the way of administering and its dosage [66,67,68,69].

#### 3.2.5. Other Substances Derived from Plants

During recent years, the analysis concerning the activity of other substances derived from plants in IBD has been conducted using numerous clinical studies. The extract containing the high concentration of anthocyanins had been administered to the patients with UC for six weeks, achieving the reduction of clinical symptoms marked in the Mayo score, the improvement of the endoscopic and histopathological images as well as the lowering the level of calprotectin in the stool. Nevertheless, after the withdrawal of therapy, the deterioration within the scope of the activity of disease and calprotectin have been observed. Other study reveals the action of silymarin in patients with diagnosed ulcerative colitis being in remission. Administered orally in the dosage of 140 mg for six months, in comparison to placebo, it causes significant improvement in biochemical parameters–lowering the ESR and rise of the hemoglobin level and it reduces the activity of a disease marked in the DAI score. In the view of the present reports, silymarin may be helpful in keeping up remission in patients with UC [70,71]. On the other hand, in children with the Crohn’s disease being in remission, the polyphenolic formula administered for ten weeks influences the marked parameters of oxidative stress positively within the scope of antioxidant defense (SOD and GPx enzymes) and the intensification of inflammation [72].

In animal models of IBD, many natural substances derived from plants are being researched according to their positive influence on inflammatory bowel diseases (e.g., rutin, diosmin, hesperidin, and others). The mentioned research constitute the basis for implementing further clinical trials with the use of plant-based components as additional therapy in IBD. Nevertheless, it is vital to take into consideration the reports about possible interactions with other drugs applied in IBD treatment, such as steroids, immunosuppressive agents, or biological therapy [84,85]. 

### 3.3. Micronutrient Antioxidants

Micronutrient antioxidants used in IBD therapy (Table 3) include inter alia vitamins E and C, reduced glutathione, and selenium.

Vitamin E is a natural substance present in plants and having a robust antioxidant activity. Moreover, it can reduce the level of free radicals, inhibit the process of lipid peroxidation and modulate the immune system. A clinical study has been conducted in a dozen of patients with a mild or moderate course of ulcerative colitis, who were administered d-alpha tocopherol rectally in the dosage of 8000 U a day as a supplementary therapy apart from conventionally applied drugs. The study revealed that after 12 weeks of treatment the improvement in all patients had been observed. The vast majority achieved remission, which was marked in the DAI score [86,93]. Unfortunately, not all scientific studies provide such unanimous results. Some of them failed to prove a positive influence of vitamin E administered orally on colitis, and marked parameters comprised the intensification of the inflammatory process and micro- and macroscopic damages. According to the fact that the part of scientific researches applying significant amounts of vitamin E reveals a positive influence on these parameters, the administered dosage should be subject to detailed analysis, as the final effect of activity, in this case, may be dependent on it [87,88]. 

Vitamin C (ascorbic acid) is a natural, exogenous, water-soluble compound with antioxidant properties. It has an omnidirectional functioning in the organism. Ascorbic acid influences, among other things, the iron absorption as well as limits cell damage caused by oxidative stress through the elimination of free radicals. Vitamin C helps to restore vitamin E and reduced glutathione levels. What is more, it boosts the adequate functioning of the immune system [94,95]. Biopsy specimens from a large intestine both in case of Crohn’s disease and ulcerative colitis reveal a distinct decrease of vitamin C levels in the tissues affected by the inflammation in comparison to healthy tissues [96]. The research on the animal models shows that vitamin C expresses an anti-inflammatory property, it decreases the DAI scale scoring system, reduces the extent of intestinal shortening and weight loss in case of colitis course. It lowers the expression of mRNA of the inflammatory process mediators as well as pro-inflammatory cytokines (iNOS, cyclooxygenase-2, TNF-α, interleukin-1β, -6, -17). Ascorbic acid also alleviates the parameters of oxidative stress through the reduction of MDA level and the enhancement of the SOD, CAT and GPx enzymes’ activity [89,90]. The research focused on the influence of a multicomponent formula (including vitamins C, A, D, E, K, fish oil, oligosaccharides, and minerals such as selenium, iron, zinc, copper) on patients with mild or moderate CU activity (rated for 3–9 points in the DAI scale). Patients qualified for the examination consumed placebo or the formula administered orally for six months. As a result, the downsizing of the total scoring in DAI scale and the histological index has been achieved. Moreover, it has been noticed that the dose of the corticosteroids needed for the proper control of the disease has been subject to reduction [97]. 

Reduced glutathione (GSH) is an organic chemical compound, one of the key antioxidants in the organism. It prevails in the organism cells in various concentrations, the highest in the hepatocytes. Under physiological conditions, glutathione occurs mainly in the reduced thiol form (GSH), while the oxidized disulfide form (GSSG) is a small part. The GSH/GSSG ratio is a very good indicator of oxidative stress. As a co-substrate of glutathione peroxidase (GPx) and glutathione S-transferase (GST), GSH participates in the reduction reactions of H_2_O_2_ and lipid peroxides as well as the coupling reactions with reactive oxidative damage products, e.g., 4-hydroxy-2-nonenal (HNE). The GSSG produced in these reactions is transformed in GSH in the reaction catalyzed by glutathione reductase (GR) in the presence of NADPH. Apart from powerful antioxidant properties, it is vital, among other things, for neutralizing the toxic substances as well as influences the immunological processes, regulates the processes of natural cell death and proliferation. There is a relation between the reduced GSH level and the dysfunctions of the chemical processes vital for its synthesis with various diseases, e.g., diabetes or cirrhosis [98,99]. GSH also plays an essential role in the transformation of pro-drug azathioprine—used in IBD treatment—into the biologically active metabolite, whereas the activity decline of glutathione-S-transferase resulting from genetic polymorphism may refer to the worsened response to the therapy using this drug. Multicausal and unclear IBD etiology dictate the search for potential causes, also among genetic factors. It has been established that the specified genotype leading to the decrease of the glutathione-S-transferase activity increases the probability of getting UC or CD considerably [100]. 

Selenium (Se) is a microelement responsible for a proper course of physiological and biochemical processes in the organism. It is vital for the synthesis of many enzymes, including glutathione peroxidase, responsible directly for the antioxidant defense of cells. Selenium supplementation exerts the immunomodulatory effect by promoting the formation of macrophages population with anti-inflammatory properties (M2 phenotype). The studies in animal models of colitis are not unanimous. On the one hand, they reveal that the application of selenium contributes to decreasing the intensity of inflammation, alleviates the course of IBD, and reduces the risk of death. Other scientific works prove that the supplementation of selenium before the induction of colitis does not affect the parameters of an inflammatory process and the course of disease significantly, whereas the supply of selenium in the acute phase of the illness may contribute even to the exacerbation of its course. A crucial factor influencing the therapeutic activity of selenium in IBD may be the severity of a disease, the dosage, the length of therapy, and also the level of selenium deficit before the beginning of symptoms, which requires further research [91,92,101,102]. 

### 3.4. Probiotics

One of the possible reasons IBD is a disturbed functioning of the immune system is because it reacts improperly with the proper microflora of the gastrointestinal tract. The phenomenon associated with IBD etiology is dysbiosis, which is based both on the decreased abundance of some bacteria and the reduced variety of bacterial flora. It is related also to the activity of the disease process because during the exacerbation of a disease the composition of a microbiome differs from the one observed during remission [103,104]. 

The research conducted in people affected with UC reveals that oral supplementation with *Bifidobacterium longum* for eight weeks lowers the clinical activity of a disease, and the severity of its course. Moreover, it improves significantly the image marked during the endoscopic examination [105]. 

During recent years the researchers have been focused on the VSL#3 preparation which constitutes a mix of 450 billion bacteria per one gram, and these bacteria belong to 8 different strains. There are reports that the treatment using VSL#3 in comparison to the group obtaining placebo contributes to maintaining remission in children affected with ulcerative colitis. According to the binding UC ECCO Guideline, VSL#3 causes the maintenance of remission in chronic pouchitis, which appears as a complication in patients who undergo the surgery of a large intestine due to UC. More and more publications emerge confirming the positive influence of this preparation, claiming that it induces remission in UC as a therapy accompanying the drugs applied conventionally. Nevertheless, ECCO Guideline currently does not formulate official recommendations related to the application of VSL#3. When it comes to Crohn’s disease, it is not advised to apply probiotics in maintaining remission [106,107,108,109,110]. 

Another strain of bacteria being intensely tested in the context of a supplementary therapy in IBD is *Escherichia coli Nissle 1917* (EcN). Meta-analyses of clinical tests conducted until now reveal that EcN prevents the return of UC in the inactive phase, and its effectiveness in preventing the recurrence of quiescent UC in comparison to 5-ASA may be similar. Currently, the therapy using EcN to induce remission is not advised. Nevertheless, there are some reports about its positive influence, especially in the forms affecting the ending sections of the gastrointestinal tract [111,112,113].

The use of probiotics as an additional treatment for IBD appears to be feasible in the near future, according to the encouraging results of studies. Nevertheless, there is a need to establish the exact dosages, route of administration, and identification of the strains that are the most effective in IBD.

## 4. Conclusions and Prospects for Future Research

IBD is a group of multifactorial, debilitating, incurable diseases, which precise etiology remains unclear. The process underlying the etiology of IBD is chronic inflammation, which is associated with oxidative/nitrosative stress and excessive production of ROS/RNS. Redox imbalance can disturb cell homeostasis and it lasts a long time can lead to its destruction. Therefore, various substances that possess antioxidant properties are now intensively studied as a possible treatment alongside classic medication or as an independent treatment option in IBD. Natural and chemical compounds with antioxidant and anti-inflammatory properties exhibit many effects that may be beneficial in UC treatment, such as scavenging of free radicals, acting as immunomodulators, increasing antioxidant defense capacity, influencing multiple signaling pathways, inhibiting pro-oxidative enzymes and cytokine levels. Based on the fact that traditional treatment in IBD exposes patients to various potentially harmful side effects, while the above-mentioned natural and chemical substances are well tolerated and have mild side effects, it is therefore one possible route and a promising direction for future studies. The research on animal models and clinical studies reveal promising results. Some substances are already being recommended in therapy, for example, VSL3# in chronic pouchitis. Despite the promising results, some studies have failed to disclose the advantageous effects of antioxidants compared to placebo, and some safety-related questions arose due to toxicity. The accurate dosage, best administration route, and new forms of delivery that overcome a clinical limitation of substances are being developed to treat IBD more effectively. Animal studies are a valuable starting point for clinical trials in humans, but so far relatively little data from these clinical trials have been obtained. In conclusion, additional well-designed clinical trials are needed to make official recommendations for therapy. 

In summary, based on the current scientific knowledge, it can be assumed that future therapy with antioxidants as an add-on therapy or independent medical options of treatment will become the strategy of choice in IBD.

## Figures and Tables

**Table 1:** Synthetic antioxidants used in IBD therapy.

Antioxidant	Clinical Studies/Animal Model	Reference
3-hydroxy-3-methylglutaryl coenzyme A (HMG-CoA) reductase inhibitors	patients with UC or CD TNBS-induced ulcerative colitis in rats DSS-induced colitis in mice	[16,17,18,19,20]
Angiotensin-converting-enzyme (ACE) inhibitors	TNBS-induced colitis in rats DSS-induced colitis in mice AA-induced ulcerative colitis in rats	[21,22,23,24]
Melatonin (MEL)	patients with UC in remission AA-induced colitis in rats DSS-induced colitis in mice TNBS-induced colitis in mice	[25,26,27,28]
N-acetylcysteine (NAC)	TNBS-induced colitis in rats	[29]
Modified superoxide dismutase (SOD)	TNBS-induced colitis in mice patients with UC DSS-induced colitis in mice	[30,31,32]
Propionyl-L-carnitine (PLC)	patients with mild-to-moderate UC or CD TNBS-induced colitis in rat TNF-α-stimulated human intestinal microvascular endothelial cells	[33,34,35]

**Table 2:** Natural antioxidants used in IBD therapy.

Antioxidant	Clinical Studies/Animal Model	Reference
Resveratrol (RSV)	TNBS-induced colitis in rats PG-PS model of Crohn’s disease in rats TNBS-induced ulcerative-colitis in rats patients with mild-to-moderate UC	[51,52,53,54,55]
Curcumin	AA-induced colitis in rats DSS-induced colitis in mice TNBS-induced colitis in rats TNBS-induced colitis in mice patients with mild-to-moderate UC	[56,57,58,59,60,61]
Quercetin (QCT)	TNBS-induced colitis in rats	[62,63]
Catechines	patients with mild-to-moderate UC human intestinal epithelial cells DSS-induced colitis in mice TNBS-induced colitis in rats	[64,65,66,67,68,69]
Anthocyanins	patients with mild-to-moderate UC	[70]
Silymarin	patients with UC in remission pediatric CD patients in remission	[71,72]

**Table 3:** Micronutrient antioxidants used in IBD therapy.

Antioxidant	Clinical Studies/Animal Model	Reference
Vitamin E (alpha-tocopherol)	patients with mild-to-moderate UC AA-induced ulcerative colitis in rats TNBS-induced colitis in rats	[86,87,88]
Vitamin C (ascorbic acid)	DSS-induced ulcerative colitis in mice	[89,90]
Selenium	DSS-induced colitis in mice	[91,92]
